# Supplementary material for: An open-source framework for large-scale, flexible evaluation of biomedical text mining systems
Source: J Biomed Discov Collab. 2008 Jan 29;3:1. doi: 10.1186/1747-5333-3-1 (PMC2276192; doi:10.1186/1747-5333-3-1)
Supplement: Additional file 1 — Evaluation platform source code. The additional file contains the software evaluation framework discussed in this paper. The most current release can be downloaded from SourceForge.net [1]. See the accompanying README file for instructions on installation and use. Also included in the distribution is a collection of UIMA wrappers for some commonly used BioNLP tools and annotated corpora. [file 1747-5333-3-1-S1.zip › bionlp_uima_repository-1.0.1/docs/api/edu/uchsc/ccp/uima/util/UIMASyntacticAnnotation_Util.html]

UIMASyntacticAnnotation\_Util (Center for Computational Pharmacology Util/UIMA API)


|  |  |  |
| --- | --- | --- |
| **Overview** \| **Package** \| **Class** \| **Tree** \| **Deprecated** \| **Index** \| **Help** | |  |
| **PREV CLASS**   NEXT CLASS | **FRAMES**    **NO FRAMES**     **All Classes** |  |
| SUMMARY: NESTED \| FIELD \| CONSTR \| METHOD | DETAIL: FIELD \| CONSTR \| METHOD |  |


---


## edu.uchsc.ccp.uima.util Class UIMASyntacticAnnotation\_Util

```
java.lang.Object
  edu.uchsc.ccp.uima.util.UIMASyntacticAnnotation_Util
```

---

``` public class UIMASyntacticAnnotation_Util extends Object ```

This class has been constructed to automate the conversion of annotations with class mention types of "token", "phrase", "clause", "sentence" into
TokenAnnotations, PhraseAnnotations, ClauseAnnotations, and SentenceAnnotations, respectively.

**Author:**
:   williamb

---

| **Field Summary** | |
| --- | --- |
| `static boolean` | `DEBUG` |


**Constructor Summary**

`UIMASyntacticAnnotation_Util()`


| **Method Summary** | |
| --- | --- |
| `static void` | `normalizeSyntacticAnnotations(org.apache.uima.jcas.JCas jcas)` |

**Methods inherited from class java.lang.Object**

`clone, equals, finalize, getClass, hashCode, notify, notifyAll, toString, wait, wait, wait`

**Field Detail**

### DEBUG

```
public static final boolean DEBUG
```

**See Also:**: Constant Field Values


**Constructor Detail**

### UIMASyntacticAnnotation\_Util

```
public UIMASyntacticAnnotation_Util()
```


**Method Detail**

### normalizeSyntacticAnnotations

```
public static void normalizeSyntacticAnnotations(org.apache.uima.jcas.JCas jcas)
```


---


|  |  |  |
| --- | --- | --- |
| **Overview** \| **Package** \| **Class** \| **Tree** \| **Deprecated** \| **Index** \| **Help** | |  |
| **PREV CLASS**   NEXT CLASS | **FRAMES**    **NO FRAMES**     **All Classes** |  |
| SUMMARY: NESTED \| FIELD \| CONSTR \| METHOD | DETAIL: FIELD \| CONSTR \| METHOD |  |


---
